# Letter to the editor in response to ‘Seasonality of the transmissibility of hand, foot and mouth disease: a modelling study in Xiamen City, China’

**DOI:** 10.1017/S095026882000059X

**Published:** 2020-03-02

**Authors:** Zehong Huang, Mingzhai Wang, Luxia Qiu, Ning Wang, Zeyu Zhao, Jia Rui, Yao Wang, Xingchun Liu, Mikah Ngwanguong Hannah, Benhua Zhao, Yanhua Su, Bin Zhao, Tianmu Chen

**Affiliations:** 1 State Key Laboratory of Molecular Vaccinology and Molecular Diagnostics, School of Public Health, Xiamen University, Fujian, People's Republic of China; 2 Xiamen Centre for Disease Control and Prevention, Xiamen City, Fujian Province, People's Republic of China; 3 Respiratory Department, Shanghai General Hospital, Shanghai, People's Republic of China; 4 Medical College, Xiamen University, Xiamen City, Fujian Province, People's Republic of China; 5 State Key Laboratory of Molecular Vaccinology and Molecular Diagnostics, Laboratory Department, Xiang'an Hospital of Xiamen University, Xiamen, Fujian, People's Republic of China

We reviewed with interest Zhao's letter regarding our article exploring the approach of calculating the effective reproduction number (*R*_eff_) [1]. We entirely agree with Zhao *et al*. that it is essential to calculate the *R*_eff_ by using the next generation matrix (NGM) approach. Actually, we also commonly used the NGM approach to calculate the reproduction number of other infectious diseases [2].

We did not provide the complex equation of *R*_eff_ from the NGM approach instead of a simplified equation in our study [1], because in Xiamen City, the values of *f*, daily *br* and daily *dr* were 0.0003 (0.03%), 2.46 × 10^−5^ and 1.24 × 10^−5^, respectively, which were much lower than those of *ω* (1/5), *γ* (1/14) and *γ*’ (1/21), respectively. We also calculated the values of *R*_eff_ by using the simplified equation we used and the two equations provided by Zhao *et al*., and we found that they were almost the same (Fig. 1).

**Fig. 1.**
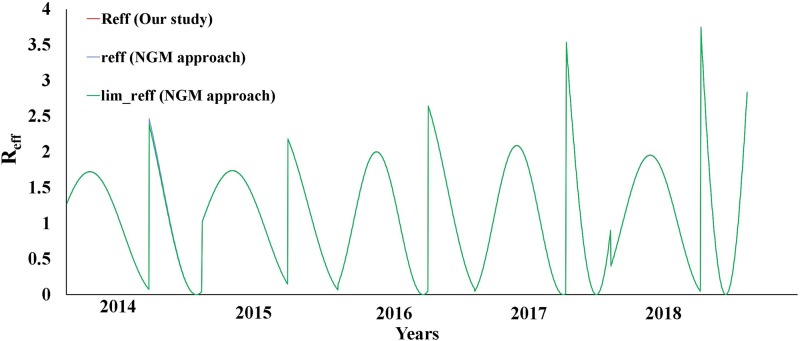
The values of *R*_eff_ calculated by three equations in Xiamen City, 2014–2018.

Therefore, we agree to use an accurate approach to estimate the transmissibility of an infectious disease. However, a simplified equation would be easier to be performed by the primary public health department than a complex one.

## References

[1] Huang Z (2019) Seasonality of the transmissibility of hand, foot and mouth disease: a modelling study in Xiamen City, China. Epidemiology and Infection 147, e327. 31884976 10.1017/S0950268819002139 PMC7006018

[2] Cui J-A (2020) Global dynamics of an epidemiological model with acute and chronic HCV infections. Applied Mathematics Letters 103, 106203.

